# Reduced Intracortical Facilitation to TMS in Both Isolated REM Sleep Behavior Disorder (RBD) and Early Parkinson’s Disease with RBD

**DOI:** 10.3390/jcm11092291

**Published:** 2022-04-20

**Authors:** Giuseppe Lanza, Filomena Irene Ilaria Cosentino, Bartolo Lanuzza, Mariangela Tripodi, Debora Aricò, Michela Figorilli, Monica Puligheddu, Francesco Fisicaro, Rita Bella, Raffaele Ferri, Manuela Pennisi

**Affiliations:** 1 Department of Surgery and Medical-Surgical Specialties, University of Catania, Via Santa Sofia 78, 95123 Catania, Italy; 2 Clinical Neurophysiology Research Unit, Oasi Research Institute—IRCCS, Via Conte Ruggero 73, 94018 Troina, Italy; rferri@oasi.en.it; 3 Department of Neurology IC and Sleep Research Center, Oasi Research Institute—IRCCS, Via Conte Ruggero 73, 94018 Troina, Italy; fcosentino@oasi.en.it (F.I.I.C.); blanuzza@oasi.en.it (B.L.); mtripodi@oasi.en.it (M.T.); darico@oasi.en.it (D.A.); 4 Neurology Unit, Department of Medical Sciences and Public Health, University of Cagliari and AOU Cagliari, Asse Didattico E., SS 554 Bivio Sestu, Monserrato, 09042 Cagliari, Italy; m.figorilli@aoucagliari.it (M.F.); puligheddu@unica.it (M.P.); 5 Sleep Disorders Center, Department of Medical Sciences and Public Health, University of Cagliari, Asse Didattico E., SS 554 Bivio Sestu, Monserrato, 09042 Cagliari, Italy; 6 Department of Biomedical and Biotechnological Sciences, University of Catania, Via Santa Sofia 78, 95123 Catania, Italy; francesco.fisicaro@phd.unict.it (F.F.); manuela.pennisi@unict.it (M.P.); 7 Department of Medical and Surgical Science and Advanced Technologies, University of Catania, Via Santa Sofia 78, 95125 Catania, Italy; rbella@unict.it

**Keywords:** cortical excitability, neurotransmitters, REM sleep behavior disorder, parkinsonian syndrome, transcranial magnetic stimulation

## Abstract

Background: a reduced intracortical facilitation (ICF), a transcranial magnetic stimulation (TMS) measure largely mediated by glutamatergic neurotransmission, was observed in subjects affected by isolated REM sleep behavior disorder (iRBD). However, direct comparison between iRBD and Parkinson’s disease (PD) with RBD is currently lacking. Methods: resting motor threshold, contralateral cortical silent period, amplitude and latency of motor evoked potentials, short-interval intracortical inhibition, and intracortical facilitation (ICF) were recorded from 15 drug-naïve iRBD patients, 15 drug-naïve PD with RBD patients, and 15 healthy participants from the right First Dorsal Interosseous muscle. REM sleep atonia index (RAI), Mini Mental State Examination (MMSE), Geriatric Depression Scale (GDS), and Epworth Sleepiness Scale (ESS) were assessed. Results: Groups were similar for sex, age, education, and patients for RBD duration and RAI. Neurological examination, MMSE, ESS, and GDS were normal in iRBD patients and controls; ESS scored worse in PD patients, but with no difference between groups at post hoc analysis. Compared to controls, both patient groups exhibited a significantly decreased ICF, without difference between them. Conclusions: iRBD and PD with RBD shared a reduced ICF, thus suggesting the involvement of glutamatergic transmission both in subjects at risk for degeneration and in those with an overt α-synucleinopathy.

## 1. Introduction

### 1.1. Background

According to the International Classification of Sleep Disorders (3rd Edition) [1], rapid eye movement (REM) sleep behavior disorder (RBD) is a “dream-enacting behaviour” parasomnia characterized by intense, usually frightening dreams, associated with different types of motor behaviors occurring in REM sleep, likely mirroring the content of dreams [2]. Isolated (or “idiopathic”) RBD (iRBD) is now viewed as an early feature of brain degenerative diseases, namely an α-synucleinopathy, such as Parkinson’s disease (PD) and other parkinsonian syndromes (such as dementia with Lewy bodies and multiple system atrophy) [3]. The REM sleep without atonia (RSWA), i.e., a decrease of muscular atonia in REM sleep, along with increased electromyographic (EMG) discharges, is the main polysomnographic (PSG) characteristics of RBD [3].

Although necessary for diagnosing RBD [1], RSWA does not seem to be the only key-feature. The search for other markers of RBD and its conversion in α-synucleinopathies has become a “cutting-edge” topic [4,5] and, accordingly, other neurophysiological aspects have been recently investigated. Among non-invasive cerebral stimulation methods, transcranial magnetic stimulation (TMS) is diffusely applied to probe in vivo neuronal circuits and cerebral networks, including prognostic information, neuromodulation applications, and neurochemical insights [6,7,8,9,10,11,12].

### 1.2. Transcranial Magnetic Stimulation (TMS): Basic Principles and Main Applications

As a neurophysiological technique, TMS was originally introduced as a non-invasive tool specifically able to evaluate the excitability of the primary motor cortex (M1) and the conductivity along the cortical-spinal tract [13], thus being applied in a number of cerebral and/or spinal disorders affecting the motor system (e.g., stroke, multiple sclerosis, motor neuron diseases, tumors, myelopathy of different etiology, etc.), even subclinically [14,15,16,17]. Nevertheless, today, TMS goes well beyond the mere assessment of the pyramidal tract, being currently used to study the pathophysiology underlying neurological and neuropsychiatric diseases, to probe in vivo and in “real-time” the excitability and plasticity of the human brain, and to assess the functioning of intracortical circuitries and callosal fibers [18,19,20,21,22]. As such, TMS is well suited for the exploration and monitoring of the electrocortical profile of several disorders, including their early stages [9,23], as well as systemic diseases involving the brain [24,25,26,27]. Finally, because TMS can assess the effects of drugs that are agonists or antagonists for specific neurotransmitters, each measure can provide the correlate of the related neurochemical activity, such as that mediated by glutamate, γ-amino-butyric acid (GABA), monoamine, or acetylcholine (the so called “pharmaco-TMS”) [28,29,30].

Technically, TMS is based on the Faraday’s law of electromagnetic induction: a transducing coil, attached to a high-voltage high-current discharge system, produces a strong time-varying and short-lasting magnetic field at right angles to the stimulation coil [31]. When coil is placed tangentially to the head, the magnetic field penetrates the skull with minimal attenuation and induces a secondary eddy current in conductive intracranial tissue [32]. The electrical field in the tissue is oriented perpendicular to the magnetic field and opposite the direction of the electrical current in the stimulation coil [33,34].

A standard examination involves bilateral recordings from distal limb muscles while the patient is seated or lying on a bed or armchair. Motor evoked potentials (MEPs), produced by stimulating the M1 at the optimum scalp position to elicit motor responses in the contralateral target muscle, are usually recorded using bipolar surface electrodes in a belly-tendon montage [33]. During TMS, the operator can control the intensity of the current flowing through the coil and, thereby, change the magnitude of the induced magnetic field and the secondarily induced electrical field within the cortex [34]. The operator can also manipulate both frequency and interstimulus interval (ISI) of the delivered stimuli, which together critically determine the effects of TMS on the targeted brain region. Accordingly, TMS can be delivered as a single pulse, as a pair of stimuli applied to the same or different brain areas, as paired cortical and peripheral stimuli, or as trains of repetitive stimuli [35].

Single TMS pulses applied to the M1 at an adequate stimulation intensity elicits a MEP in the contralateral target muscle [36]. Both MEP latency and central motor conduction time (CMCT) are considered indexes of integrity of the cortical-spinal pathway, whereas the MEP amplitude (i.e., the peak-to-peak size of the motor response) mainly reflects the excitation state of output cells in the motor cortex, nerve roots, and peripheral motor tracts, till the muscles [35]. MEP latency is the time interval between the administration of TMS over the M1 and the MEP onset from the contralateral muscle; as such, it reflects a total conduction time, accounting for both central and peripheral nervous system conductivity, as well as for neuromuscular junctions and muscles; conversely, as obtained as the latency difference between the MEPs induced by stimulation of the motor cortex and that evoked by spinal (motor root) stimulation, the CMCT reflects the integrity of the cortical-spinal tract only, from the upper to the lower motor neurons [37].

The resting motor threshold (rMT), according to the latest recommendations of the International Federation of Clinical Neurophysiology [35], is considered as a global parameter of brain excitability, since it is a compound measure of the membrane excitability of cortical-spinal neurons, neural inputs into pyramidal cells within the cortex, and spinal motor neurons, neuromuscular junctions, and muscles [38]. The administration of a suprathreshold TMS pulse to the M1 during a tonic voluntary contraction of the contralateral muscles suppresses the EMG activity in those muscles for a few hundred milliseconds [39]. This phenomenon, called contralateral cortical silent period (cSP), can be exploited to functionally measure some intracortical inhibitory circuits, mainly mediated by GABA-B transmission, as consistently demonstrated by previous pharmaco-TMS evidence [40].

Inhibitory and excitatory interneuronal activity within the human cortex can be non-invasively explored by using the paired-pulse TMS paradigm [41,42]. The conventional protocol uses a “conditioning stimulus” (CS, subthreshold) followed by a “test stimulus” (TS, suprathreshold). By varying the intensity of the CS and the ISI between the pair of TMS pulses, a number of measures of intracortical interneuronal function and interaction can be obtained. For instance, at ISI of 1–4 ms, the CS suppresses the MEP amplitude, a phenomenon called short-latency intracortical inhibition (SICI) [41], whereas at a longer ISI (7–20 ms), the stimulus results in an enhanced MEP response, called intracortical facilitation (ICF) [42]. The underlying mechanisms are thought to reflect distinctive neurochemical circuits: while SICI is likely mediated by the GABA-A interneuron activity [43,44], ICF seems to be a more complex phenomenon [45], although it is thought to be mainly produced by the activation of glutamatergic neurons [46].

TMS can be also used to test the sensory-motor interaction within specific cortical areas. For instance, the short-latency afferent inhibition (SAI) of the MEP reflects the sensory stimuli-mediated inhibitory modulation of the M1 [47]. This effect depends on the time that elapses between the peripheral nerve electrical stimulus and the TMS pulse, and it typically occurs at an ISI of 20 ms [48]. SAI may represent the neurophysiological correlate of central cholinergic activity, because it is reduced or abolished by the muscarinic receptor antagonist scopolamine [43] and it is positively modulated by acetylcholine [49].

Finally, TMS allows to study synaptic plasticity at different levels. Among the most adopted protocols, the paired-associative stimulation (PAS) consists of a slow-rate repetitive low-frequency nerve stimulation combined with the TMS over the contralateral M1; it has been shown to induce plastic changes of excitability within the motor cortex [50,51]. In the repetitive TMS (rTMS) paradigms, trains of TMS pulses at the same intensity are applied to a single brain area at a given frequency, that can transiently influence the function of stimulated and connected brain areas, mainly depending on the frequency of stimulation [52]. Generally, low-frequency rTMS (stimulus rates ≤1 Hz) induces inhibitory effects on motor cortical excitability, thus inducing the so-called “reversible virtual lesion” [53], while high-frequency rTMS (5–20 Hz) usually promotes an increased excitability [54].

### 1.3. Rationale and Aim

Regarding TMS, several investigations have been performed to assess the electrophysiological profile of motor cortex excitation, synaptic plasticity, and neural connectivity in sleep and movement disorders [55,56,57,58,59,60,61,62,63,64,65,66,67]. Conversely, up to now, a couple of studies only have applied TMS in iRBD. A first study [68] observed changes of short-latency afferent inhibition in these patients, a result which supports the possibility of cholinergic impairment in those patients who develop cognitive decline. This finding has been confirmed by a subsequent study [69] in subjects with RBD and PD, that may be considered as the correlate of cholinergic dysfunction underlying the well-known impairment of cognition described in parkinsonian syndromes. The researchers suggested that loss of cholinergic neurons may contribute to non-motor symptoms of PD, including the possibility that RBD increases the risk of cognitive deterioration of these subjects [69].

In the third, more recent, investigation in iRBD subjects without any movement or cognitive disorder [70], we found that changes in ICF and, to a lesser extent, SICI, might precede the onset of a future neurodegeneration. Recently, also a EEG investigation may strengthen these findings: EEG in REM sleep indicated mild but relevant changes in the electrocortical physiology of iRBD subjects [71]. Namely, in drug-naïve patients than healthy controls, the normalized power values showed a less pronounced REM-related decrease of power in all bands with frequency <15 Hz, as well as an increase in the β band that negatively correlated with muscle atonia; of note, in patients treated with clonazepam, there was a partial return of all bands toward the normal values. These findings may indicate the occurrence of a prodromal phase of neurodegeneration in iRBD [71].

To date, however, a direct comparison between iRBD and RBD in the context of an overt extrapyramidal syndrome (i.e., PD) is lacking. Here, we applied TMS in drug-naïve subjects with iRBD and in those affected by PD with RBD, both age-matched with healthy participants. We hypothesize that both patient groups might show a comparable electrocortical pattern at the TMS level.

## 2. Materials and Methods

### 2.1. Participants and Assessment

Fifteen de novo subjects with clinically- and PSG-confirmed iRBD (11 men; age, median: 67 years, range: 60–69; disease duration, median: 3 years, range: 1–4) and 15 de novo patients affected by PD with RBD (8 men; age, median: 70 years, range: 63–79; disease duration, median: 3.5 years, range: 2–5) were consecutively enrolled from the Sleep Research Centre, Oasi Research Institute—IRCCS of Troina (Italy). RBD was diagnosed according to the current diagnostic criteria of the American Academy of Sleep Medicine, 3rd edition of the International Classification of Sleep Disorders [1], whereas diagnosis of PD was based on the current clinical diagnostic criteria of the Movement Disorder Society (MDS) [72]. Both groups of patients were age-matched with 15 healthy participants (10 men; age, median: 65 years, range: 60–70).

Participants were excluded if: <18 years old; presence or history of any psychiatric diseases (such as depressive, bipolar, or psychotic disorders), nervous system diseases other than PD (such as atypical parkinsonian syndromes, seizures, cerebrovascular diseases, dementia, traumatic brain or spinal injury, multiple sclerosis, peripheral nervous system diseases); other sleep disorders, e.g., restless legs syndrome, periodic limb movements (index >15 per hour), obstructive sleep apnea syndrome, insomnia, narcolepsy, sleep-wake rhythm disorders; acute or non-compensated illnesses; clinical or drug-associated conditions causing cognitive or mood dysfunction; alcohol/illicit drug use or history; use of psychoactive drugs or any medication capable of modulating brain excitation [40,73]; Mini Mental State Examination (MMSE) [74] <24, Geriatric Depression Scale (short form) (GDS) [75] >5, and MDS Unified PD Rating Scale-part III (UPDRS-III) [76] >0 (for iRBD and controls only); conditions excluding magnetic resonance imaging (MRI) or TMS.

Demographic and clinical evaluation included sex, age, educational level, handedness, medical and family history, neurological and general exams. The right-handedness of each participant was verified by means of the Edinburgh Handedness Inventory [77]. None reported relevant co-morbidities; family history was positive for RBD and PD in two and four patients, respectively. Patients were not on any medication for PD or RBD, and none of the participants was on psychoactive medication.

Preliminarily, UPDRS-III was administered in all patients to rule out clinical motor manifestations in iRBD and to quantify the motor impairment in PD. Cognitive screening tool (MMSE), assessment of depressive symptomatology (GDS), and subjective sleepiness through the Epworth Sleepiness Scale (ESS) [78] were quantified as well. Conventional EEG was conducted to exclude predisposition to seizure. Additionally, to rule out a potential peripheral component affecting the cortical excitation state, a standard electroneurography of the right ulnar nerve was carried out, that resulted normal in every participant. A 1.5 T MRI of the brain, performed in each patient, was normal.

Local Ethics Committee approved this study (prot. 2018/07/18/CE-IRCCS-OASI/14; date of approval 18 July 2018), which was performed in agreement with the Declaration of Helsinki of 1964 and subsequent amendments. Each subject provided written informed consent prior to the enrolment, after explanations and full acceptance of all procedures.

### 2.2. Calculation of the REM Sleep Atonia Index (RAI)

Conventional overnight PSG was carried out in each patient, including Submentalis muscle EMG (bipolar derivations, two electrodes positioned 3 cm apart and fixed with collodion-soaked gauze pad). Impedances were maintained <10 KΩ, typically <5 KΩ.

Regarding quantitative RAI calculation, an automatic established algorithm was adopted [79,80]. The EMG signal from the Submentalis muscle was digitally band-pass filtered at 10–100 Hz (notch filter: 50 Hz) and rectified. Then, every epoch of REM sleep analyzed was divided in 30 mini-epochs of 1 s each. The average amplitude of the rectified EMG signal was thus calculated for every mini-epoch obtained. After a reducing noise procedure [81], the average amplitude of the signal in each mini-epoch was utilized to calculate the mini-epochs percentage falling within the following 20 amplitude (amp) classes (μV): amp ≤ 1, 1 < amp ≤ 2, …, 18 < amp ≤ 19, and amp > 19. Muscular atonia was supposed to be reflected by high values of the first class (amp ≤ 1), while tonic and phasic activations were supposed to increase values of the other classes [82,83,84]. Subsequently, RAI was obtained, summarizing in a single value the preponderance degree of the first class in REM sleep: RAI = amp ≤ 1/(100 − 1 < amp ≤ 2). This index can mathematically vary from 0 (lack of mini-epochs with amp ≤ 1), i.e., total lack of EMG atonia, to 1 (all mini-epochs with amp ≤ 1), i.e., normal EMG atonia. The algorithm was run blind to the patient’s group allocation and no manual change of the parameters was possible.

### 2.3. Transcranial Magnetic Stimulation (TMS)

TMS was carried out through a high-power magnetic stimulator Magstim 200^2^ (Magstim Co., Whitland, Dyfed, UK). A “figure-of-eight” coil (external diameter: 90 mm) was positioned on the M1 at the optimal location of the scalp to generate MEPs in the contralateral First Dorsal Interosseous (FDI) muscle of the dominant side. Given the right-handedness of all participants, the M1 of the left hemisphere was stimulated, with the current induced running in a posterior-anterior flow, as required [35]. EMG was registered by means of disposable self-conductive and self-adhesive silver/silver-chloride surface electrodes. Active electrode was positioned on the FDI muscle belly and reference distally at the index finger metacarpal-phalangeal joint, whereas ground electrode was placed over the wrist dorsal surface. MEPs were filtered (bandwidth 3–3000 Hz) and amplified [35].

As internationally defined [35], rMT was considered as the minimum stimulation intensity capable of generating resting MEP of at least 50 µV in amplitude in 5 of 10 trials. CMCT was estimated by subtracting the conduction time along the peripheral nerve, after magnetic stimulation of cervical roots, from the cortical-muscular latency of MEP after active moderate muscular contraction, with an intensity of stimulation equal to 130% of the individual rMT. MEP amplitude, measured from peak to peak and obtained during muscle activation, was also recorded. Contralateral cSP was studied during ~50% of the maximal voluntary tonic activation of the FDI muscle, obtained after single pulses TMS at 130% of the rMT; mean duration of 10 rectified cSP recordings was then estimated [35].

Paired-pulse TMS was carried out with the same coil and through the elicitation of magnetic stimuli from two Magstim 200^2^ stimulators by using a “BiStim” module to connect each other. ICF and SICI were recorded through the above-describe CS-TS protocol [41,42]: the CS was equal to 80% of the subject’s rMT and the TS to 130%. This setting, indeed, allows to elicit MEPs in the FDI at rest with a size measured from a peak to peak of ~1 mV. ISIs used in this study were 3 and 10 ms, since they are considered the optimal intervals capable of producing a clear suppression and facilitation of the MEP response, respectively [18,41,42]. In fact, SICI at 3 ms has been shown to represent the best post-synaptic inhibitory potential mediated by the GABA-A activity [44,85], as well as 10 ms was compatible with the most suitable recruitment of the facilitatory (mostly glutamatergic) post-synaptic responses reflecting ICF [46]. For both ISIs, 10 trials were randomly recorded, with an interval of 8 s between each stimulation. Responses were calculated as the ratio between the amplitude of MEP produced after the paired-stimulation and that obtained after the “test” stimulus only [41,42]. A constant visual-audio high-gain EMG feedback helped participants in keeping total muscle relaxation.

All procedures were performed while participants seated in a dedicated armchair, in the same Lab, experimental conditions, and equipment and by the same experienced operators, at the similar daytime (~10:00–11:30 a.m.). Data were collected on the Lab computer and stored through a dedicated software for the analysis off-line [86].

### 2.4. Statistical Analysis

Since some variables had non-normal distribution, any difference between the continuous variables in the different groups of participants (iRBD, PD+RBD, controls) were assessed through the non-parametric Kruskal-Wallis ANOVA, followed by the Mann-Whitney test for independent datasets, utilized as a post hoc analysis for comparing each pair of groups, as appropriate. Frequencies were compared through the Chi-square test. *p*-values were considered statistically significant when <0.05.

## 3. Results

All participants completed the procedure and did not report any adverse event or undesired effect/discomfort. Table 1 shows the demographic and clinical features of all participants included in this study.

Groups were similar for age, sex, education, MMSE, and GDS, as well as patients for both RBD duration and RAI.; the same holds true for sex distribution (chi-square 1.36, *p* = 0.507). ESS scored worse in PD patients (*p* = 0.048), although the median value was still within the normal limit (5.0, range 2.0–7.0) and significant differences were not found between groups at *post hoc* analyses. The neurological examination was normal in all iRBD patients and healthy controls, whereas PD subjects overall showed mild-to-moderate motor symptoms based on the UPDRS-III [87] (<33) and disability based on the modified Hoehn & Yahr staging scale [88] (≤2.5).

Table 2 shows that all single-pulse TMS measures were comparable between the groups. At paired-pulse TMS, the two patient groups revealed a significantly decreased ICF (iRBD: 0.7, range 0.1–1.0; PD: 0.2, range 0.1–1.3; controls: 1.9, range 1.4–2.3; *p* = 0.0001) with respect to control subjects, but without differences between iRBD and PD with RBD.

## 4. Discussion

### 4.1. Main Findings and Translational Implications

To our knowledge, this is the first TMS investigation which has directly compared iRBD patients with those with PD and RBD. The main finding is that both groups of patients shared a reduced ICF to paired-pulse TMS, thus suggesting the involvement of glutamatergic transmission both in subjects at risk for neurodegeneration and in those with an overt α-synucleinopathy. This has allowed to support our experimental hypothesis, i.e., that both patient groups would have showed a similar electrocortical pattern to TMS, although the mechanisms underlying this result seem to be rather complex, given also the paucity of previously published data.

It should be preliminarily noted that no difference between both patient groups and healthy subjects was noted for single-pulse TMS measures (i.e., rMT, MEP amplitude and latency, CMCT, and cSP), thus suggesting that both global motor cortex excitation and cortico-spinal conduction may be not involved in both PD and RBD, thus supporting previous observations [89]. More interestingly, however, these results reveal that TMS may unveil subtle modifications in the M1 pathophysiology and, therefore, it can be more helpful than clinical observation alone in detecting even early phases of a neurodegenerative disorder. Accordingly, the presence of RBD in the context of PD has been linked more with a neocortical, thalamic, and limbic denervation than dopaminergic nigro-striatal degeneration, thus possibly suggesting a diverse pathophysiology and distinctive etiology compared to PD without concomitant or preceding RBD [90].

On the other hand, changes in the pattern of cortical excitability to TMS have been consistently reported in PD, thus expanding our knowledge of cortical excitability and neural plasticity in these patients as well [91]. Namely, ICF has been found to be enhanced in the off-medication state [92], although this was not the case of our patients, who were all drug-naïve at the time of the examination. An earlier study did not observe difference of ICF between patients and controls [93], whereas, more recently, other researchers have found it to be significantly reduced [61,62]. Moreover, high-frequency (excitatory) rTMS did not facilitate [94] and the low frequency (inhibitory) did not suppress the MEP amplitude [95] in these patients, thus supporting the findings of impaired facilitation and plasticity in PD. Similarly, MEPs were not facilitated in these patients after PAS protocols [7], suggesting an impairment of the spike-timing dependent plasticity in the motor cortex. In a more recent study comparing PD patients with and without RBD, the authors found that those with RBD had greater SICI and reduced ICF, likely indicating distinctive pathophysiological processes underlying PD with or without RBD also at the TMS level [96]. Finally, in a very recent study using TMS to assess cortical excitability changes in PD with varying degree of cognitive impairment [97], patients had graded reduction in ICF as the disease progressed from PD without cognitive decline through PD with mild cognitive impairment, to an overt PD-dementia. Therefore, the early changes of ICF we observed in still cognitively intact RBD patients, with or without PD, might be considered as an early, preclinical, marker of incipient cognitive dysfunction. Taken together, these findings suggest an overall imbalance of intracortical circuits in PD and a defective facilitatory cortical inputs, particularly for movement execution and cognitive functioning.

This electrophysiological evidence seems to converge on the possibility that RBD may alter a global neurochemical network in different regions of the central nervous system (CNS) in a broader manner than the atonia-generating circuitry within the brainstem alone, including also the cerebral cortex (i.e., M1) by means of specific ascending projections (i.e., the thalamo-cortical pathway and the reticular formation) [68]. If the evidence of glutamatergic impairment is due to a direct lesion of the cortex or, indirectly, through a damage arising from the brainstem areas regulating the REM sleep, and subsequently projecting to the cortex, is not established yet. Nevertheless, both neuroimaging and histopathologic findings have demonstrated the involvement of a number of brainstem areas and related neurochemical transmissions in RBD [98]. All these regions are known to diffusely project into cerebral cortical areas and, as a consequence, any alteration of this complex framework can justify the possibility of a cortical dysfunction in RBD [70]. This hypothesis has been also validated by earlier studies linking cortical thinning and phenoconversion of RBD [99]. Furthermore, diffusion tensor imaging and volumetric MRI did not reveal any significant difference in terms of loss of neurons within the pedunculopontine nucleus based on RBD symptoms, thus indicating that pontine cholinergic disruption alone cannot be enough to explain the whole clinical picture of RBD patients [100].

In this context, our understanding on the neuronal circuits at the basis of REM sleep physiology and mechanisms underlying RSWA largely comes from preclinical, histopathological, and neuroimaging research. Indeed, any damage of the brainstem, e.g., secondary to neurodegenerative, demyelinating, neoplastic, or vascular damage, can induce RSWA, thus leading to a “lesional” RBD [101,102,103,104]. Shortly, the sublaterodorsal tegmental nucleus (SLD) and, particularly the glutamatergic neurons called “REM-on”, has been identified as the cerebral area triggering the atonia during the REM stage of sleep through the stimulation of the ventromedial medulla (VMM), as well as the interneurons within the spinal level directly inhibiting motor neurons through specific GABA- and glycine-mediated projections. VMM and SLD constitute the “REM sleep atonia circuit” and, as such, their damage prevent paralysis during REM sleep [98].

Overall, both preclinical and clinical studies suggested that RBD may result from a disruption of a wide network underlying the atonia during the REM sleep, that represents the basis for a model of dynamic interactions between the brainstem and both upper and lower CNS regions [105]. Additionally, the evidence of EEG instability of REM sleep in RBD subjects [71] should guide the research towards additional elucidation on how the cortex and the brainstem interact. Taken together, these findings add further support the modern pathophysiological definition of RBD as a complex network disorder of the REM sleep and not simply as a brainstem and acetylcholine only pathology [105,106].

Finally, glutamate activity or its modulation may emerge as a novel therapeutic target for both iRBD and PD. Their enhancement by pharmacological or neuromodulation interventions (e.g., repetitive TMS-based protocols) might represent innovative “TMS-guided” therapeutical strategies [107]. In this scenario, since it is mandatory a validation of objective indexes able to stratify at risk subjects or those who can be candidate for treatments, the application of non-invasive cerebral stimulation methods as a probing tool of neurochemical circuit integrity will be of scientific and clinical relevance [108]. Moreover, since ICF is very sensitive to different pharmacological agents [40,46,73], it could also be useful to monitor these patients and follow-up the treatment efficacy.

### 4.2. Limitations and Future Outlooks

Some limitations should be mentioned. First, as usual in TMS studies, the small sample size. It is likely indeed that, though all drug-naïve and homogeneous for clinical, demographics, and hypnological characteristics, the relatively small number of participants was not sufficient to reach further statistically significant differences.

Second, the current study did not include PD patients without RBD and, therefore, we cannot infer on the possible role of iRBD in the further phenotypization of PD.

Third, this investigation was carried out during wakefulness, which is obviously a very different condition from sleep; nevertheless, as earlier reviewed [60], almost all previous reports (even the most recent studies), were conducted in the awake state likely because of procedural or technical difficulties.

Lastly, though glutamate is believed to mostly mediate ICF [18], this is actually a more complex measure, being other transmission systems (e.g., noradrenalin, acetylcholine, and dopamine) [40,46,73] possibly involved, although their functioning was not assessed in the present study. Moreover, while growing evidence points out the cortical origin of ICF [45], its neurochemical correlates and underlying microcircuits are debated yet [109]. However, ICF did not seem to be associated with the N-methyl-D-aspartate (NMDA) receptor activity, given that NMDA antagonist ketamine does not inhibit it [110].

Research agenda should also consider studies including the exploration of the emotional reactivity and mood disorders in patient with RBD and PD. In this context, evidence indicates a clear relationship between temperament and depression in PD patients under dopaminergic treatment, and different temperament straits have been associated with stress and anxiety and depression [111], which, as RBD, are typical non-motor symptoms of these patients. Moreover, rehearsal of negative memories, often associated with depression and stress, is able to alter corticospinal excitability and plasticity [112,113].

## 5. Conclusions

TMS, together with clinical, sleep-related, and imaging data, can provide additional hints on the pathomechanisms underlying the cortical involvement in both PD and RBD and possibly open the way towards innovative therapeutical targets. Follow-up examinations are needed to confirm if the TMS changes observed at this stage will correlate with clinical evolution and the response to treatment.

## Figures and Tables

**Table 1:** Demographic and clinical characteristics of the three groups of participants.

	1. Controls		2. iRBD		3. PD with RBD		Kruskall-Wallis ANOVA		Mann-Whitney *p*		
**Variable**	*median*	*IQR*	*median*	*IQR*	*median*	*IQR*	*H(2.45)*	*p*	*1* vs. *2*	*1* vs. *3*	*2* vs. *3*
Age, years	65.0	60.0–70.0	67.0	60.0–69.0	70.0	63.0–79.0	2.128	NS	-	-	-
Education, years	8.0	5.0–13.0	8.0	5.0–13.0	8.0	5.0–13.0	0.019	NS	-	-	-
RBD duration, years	-	-	3.0	1.0–4.0	3.5	2.0–5.0	-	-	-	-	NS
MMSE	28.0	26.7–29.5	27.0	26.0–29.0	27.3	24.7–28.7	2.257	NS	-	-	-
ESS	2.0	0.0–5.0	4.0	3.0–9.0	5.0	2.0–7.0	6.086	0.048	NS	NS	NS
GDS	3.0	0.0–4.0	1.0	0.0–4.0	2.0	1.0–5.0	1.058	NS	-	-	-
RAI	-	-	0.782	0.731–0.886	0.853	0.474–0.939	-	-	-	-	NS

Legend (*in alphabetical order*): ESS, Epworth Sleepiness Scale; GDS, Geriatric Depression Scale (short form); IQR, interquartile range; iRBD, isolated REM sleep behavior disorder; MMSE, Mini Mental State Examination; NS, not significant; PD, Parkinson’s disease; RAI, REM sleep atonia index; RBD, REM sleep behavior disorder.

**Table 2:** Single and paired-pulse TMS comparison between patient groups and healthy controls.

	1. Control Subjects		2. iRBD Patients		3. PD with RBD Patients		Kruskall-Wallis ANOVA		Mann-Whitney *p*		
**Variable**	*median*	*IQR*	*median*	*IQR*	*median*	*IQR*	*H(2.45)*	*p*	*1* vs. *2*	*1* vs. *3*	*2* vs. *3*
rMT, %	43.0	40.0–47.0	44.0	38.0–46.0	46.0	39.0–48.0	1.266	NS	-	-	-
cSP, ms	71.8	51.0–80.5	69.0	64.8–78.9	69.5	51.8–86.2	0.381	NS	-	-	-
MEP latency, ms	21.2	20.2–21.9	21.3	20.3–21.9	21.2	20.9–22.3	0.224	NS	-	-	-
MEP amp, mV	1.9	1.4–3.0	2.3	1.6–3.0	2.9	1.7–3.8	0.916	NS	-	-	-
CMCT, ms	6.3	5.9–7.6	6.4	6.0–7.7	6.4	5.4–7.6	0.628	NS	-	-	-
MEP amp (ISI 0 ms)	1.1	0.8–1.6	1.0	0.7–1.5	0.9	0.4–2.0	0.233	NS	-	-	-
SICI, ratio (ISI 3 ms)	0.2	0.1–0.3	0.6	0.1–1.4	1.0	0.1–1.1	3.918	NS	-	-	-
ICF, ratio (ISI 10 ms)	1.9	1.4–2.3	0.7	0.1–1.0	0.2	0.1–1.3	19.145	0.0001	0.0003	0.0002	NS

Legend (*in alphabetical order*): amp, amplitude; CMCT, central motor conduction time; cSP, cortical silent period; ICF, intracortical facilitation; IQR, interquartile range; iRBD, isolated REM sleep behavior disorder; ISI, interstimulus interval; M, median value; MEP, motor evoked potential; NS, not significant; PD, Parkinson’s disease; RBD, REM sleep behavior disorder; rMT, resting motor threshold; SICI, short-interval intracortical inhibition.

## Data Availability

Data is contained within the article.
